# Current Status and Trends in Peptide Receptor Radionuclide Therapy in the Past 20 Years (2000–2019): A Bibliometric Study

**DOI:** 10.3389/fphar.2021.624534

**Published:** 2021-04-27

**Authors:** Xiaojing Lu, Cuncun Lu, Yongjie Yang, Xiangfen Shi, Haibo Wang, Nan Yang, Kehu Yang, Xiaojian Zhang

**Affiliations:** ^1^ Department of Pharmacy, The First Affiliated Hospital of Zhengzhou University, Zhengzhou, China; ^2^ Evidence-Based Medicine Center, School of Basic Medical Sciences, Lanzhou University, Lanzhou, China; ^3^ Institute of Basic Research in Clinical Medicine, China Academy of Chinese Medical Sciences, Beijing, China; ^4^ School of Integrated Traditional Chinese and Western Medicine, Gansu University of Chinese Medicine, Lanzhou, China

**Keywords:** bibliometrics, peptide receptor radionuclide therapy, VOSviewer, CiteSpace, R-bibliometrix

## Abstract

**Background:** Peptide receptor radionuclide therapy (PRRT) is an emerging therapeutic option for the treatment of neuroendocrine tumors (NETs), and the number of publications in this field has been increasing in recent years. The aim of the present study was to present the research status and summarize the key topics through bibliometric analysis of published PRRT literature.

**Methods:** A literature search for PRRT research from 2000 to 2019 was conducted using the Science Citation Index Expanded of Web of Science Core Collection (limited to SCIE) on August 4, 2020. The VOSviewer, R-bibliometrix, and CiteSpace software were used to conduct the bibliometric analysis.

**Results:** From 2000 to 2019, a total of 681 publications (523 articles and 158 reviews) were retrieved. Annual publication outputs grew from three to 111 records. Germany had the largest number of publications, making the largest contribution to the field (*n* = 151, 22.17%). Active cooperation between countries/regions was observed. Kwekkeboom from the Erasmus Medical Center is perhaps a key researcher in the field of PRRT. The *European Journal of Nuclear Medicine and Molecular Imaging* and *Journal of Nuclear Medicine* ranked first for productive (*n* = 84, 12.33%) and co-cited (*n* = 3,438) journals, respectively. Important topics mainly included matters related to the efficacy of PRRT (e.g., ^90^Y-dotatoc and ^177^Lu-dotatate), the long-term adverse effects of PRRT (e.g., hematologic and renal toxicities), standardization of NETs and PRRT in practice, the development of medical imaging techniques, and the individual dose optimization of PRRT.

**Conclusion:** Using bibliometric analysis, we gained deep insight into the global status and trends of studies investigating PRRT for the first time. The PRRT field is undergoing a period of rapid development, and our study provides a valuable reference for clinical researchers and practitioners.

## Introduction

Peptide receptor radionuclide therapy (PRRT) is a molecularly targeted radiation therapy involving the systemic administration of a radiolabeled peptide (e.g., ^90^Y-dotatoc and ^177^Lu-dotatate), which is designed to target receptors overexpressed on tumors (e.g., somatostatin receptor subtype 2) with high affinity and specificity (Zaknun et al., 2013). At present, PRRT is mainly used for the treatment of metastatic or inoperable neuroendocrine tumors (NETs). As an emerging therapeutic option for NETs, the efficacy of PRRT with radiolabelled somatostatin analogs, such as ^90^Y-dotatoc and ^177^Lu-dotatate, is encouraging (Imhof et al., 2011; Pfeifer et al., 2011; Kam et al., 2012). For example, a multinational phase III randomized clinical trial evaluated the efficacy of PRRT in patients with advanced, progressive, somatostatin receptor–positive midgut NETs. Two hundred and twenty-nine patients were randomized to either high-dose long-acting release octreotide therapy or ^177^Lu-dotatate. The study showed that treatment with ^177^Lu-dotatate was associated with significant patient benefit, which paved the way for the approval of Lutathera^®^ (^177^Lu-dotatate) in the United States and Europe (Strosberg et al., 2017; Hennrich and Kopka, 2019). Encouraging results have also been reported from other studies that evaluated ^177^Lu-dotatate for the treatment of other subtypes of NETs (Sansovini et al., 2013; Brabander et al., 2017). However, the side effects of PRRT also need to be considered, such as hematologic and renal toxicities (Bodei et al., 2008; Sabet et al., 2013; Sonbol et al., 2020). Furthermore, compared with ^177^Lu, ^90^Y-labeled peptides are more likely to result in higher levels of overall toxicity. Additionally, studies have also reported treatments that combine ^177^Lu and ^90^Y (Kunikowska et al., 2011; Seregni et al., 2014) as well as the application of PRRT in combination with other therapies, such as chemotherapy (Kong et al., 2014; Claringbold and Turner, 2016; Kong et al., 2017).

Bibliometrics, a widely accepted research method based on statistical and visualization techniques, helps to depict the knowledge structures and developmental trends of a specific field (Tran et al., 2019; Ke et al., 2020). Generally speaking, a bibliometric analysis usually includes the following steps: putting forward research questions, searching databases (e.g., Web of Science and Scopus), collecting and analyzing data, drawing maps, reporting results, and submitting the manuscript (Ke et al., 2020). To date, bibliometrics has been carried out in a wide range of research topics (Zhang et al., 2015; Yang et al., 2018; Tran et al., 2019; Ke et al., 2020), such as health care, environmental science, and energy management. For example, Tran et al. employed this method and found that artificial intelligence has been applied to the field of health care for a wide range of purposes (Tran et al., 2019). Several bibliometric tools have been developed and are now being used frequently, which include VOSviewer, CiteSpace, BICOMB, and BibExcel. The application of these tools allows researchers to evaluate the current state of a subject and identify hotspots with ease, especially for beginners and non-professional researchers.

In recent years, a number of papers focusing on PRRT have been published; however, to the best of our knowledge, there have not been any studies that provide an overview of PRRT from the perspective of bibliometrics. Therefore, we performed a bibliometric analysis to gain a comprehensive view of the research trends concerning PRRT from multiple aspects, including number of publications per year, productive countries/regions, important journals, key references, and research foci. Our aim is to provide a reference for clinical researchers and practitioners.

## Methods

### Data Source

On August 4, 2020, we conducted a literature search using the Web of Science Core Collection (WoSCC), limited to Science Citation Index-Expanded (SCIE), to identify PRRT-related publications (only “article” and “review”) from the past two decades (from 2000 to 2019) with no language restriction. Our search strategy was adapted from a recently published systematic review (Sonbol et al., 2020) as follows: TOPICS = “peptide receptor radionuclide therapy” OR “peptide receptor radionuclide therapies” OR “lu-dotatoc” OR “lutetium-dota” OR “lu-dotatate” OR “y-dotatate” OR “y-dotatoc” OR “tyr3-octreotide.” All retrieved records were downloaded on August 4, 2020, and imported into bibliometric tools for further analysis.

### Statistical Analysis

The annual output (number of publications per year), publication languages, and document types of PRRT research were analyzed by the online data analysis function (“Analyze Results”) of the WoSCC. Journal impact factors were obtained from the 2019 Journal Citation Reports (Clarivate analytics, Philadelphia, PA, United States). VOSviewer (1.6.15) was used to identify productive countries/regions (if one publication was completed by more than one country/region, the publication was assigned equally to all participating countries/regions), journals, main co-cited journals (if a journal has changed its name, they will be considered as different journals when counting), and key references. The desired bibliometric maps were created. On the VOSviewer maps, different bubbles represent elements (countries/regions, journals, and references), while the size of the bubbles represents the number of publications or co-occurrence frequency. A line between two bubbles reflects the relationship, and the thickness of the lines reflects the strength of the relationship between the elements (van Eck and Waltman., 2010; Ke et al., 2020). In this research, the VOSviewer parameters were set as follows: the counting method was full counting, and the threshold (T) of the elements was dependent on the corresponding element. The geographical distribution map of countries/regions was created using R-bibliometrix (Aria and Cuccurullo., 2017). CiteSpace (5.6. R5) is an application for visualizing and analyzing trends and patterns in scientific literature, which was used to construct the dual-map overlay for journals and detect references with strong citation burstness to identify key topics (Hou et al., 2018; Ke et al., 2020). The parameters of CiteSpace were set as follows: link retaining factor (LRF = 3), look back years (LBY = 8), e for top N (*e* = 2), time span (2000–2019), years per slice (1), links (strength: cosine, scope: within slices), selection criteria (Top N: top 50), and minimum duration (MD = 5). The data were managed using Microsoft Office Excel 2019 (Redmond, Washington, United States).

## Results

### Annual Output

**FIGURE 1:**
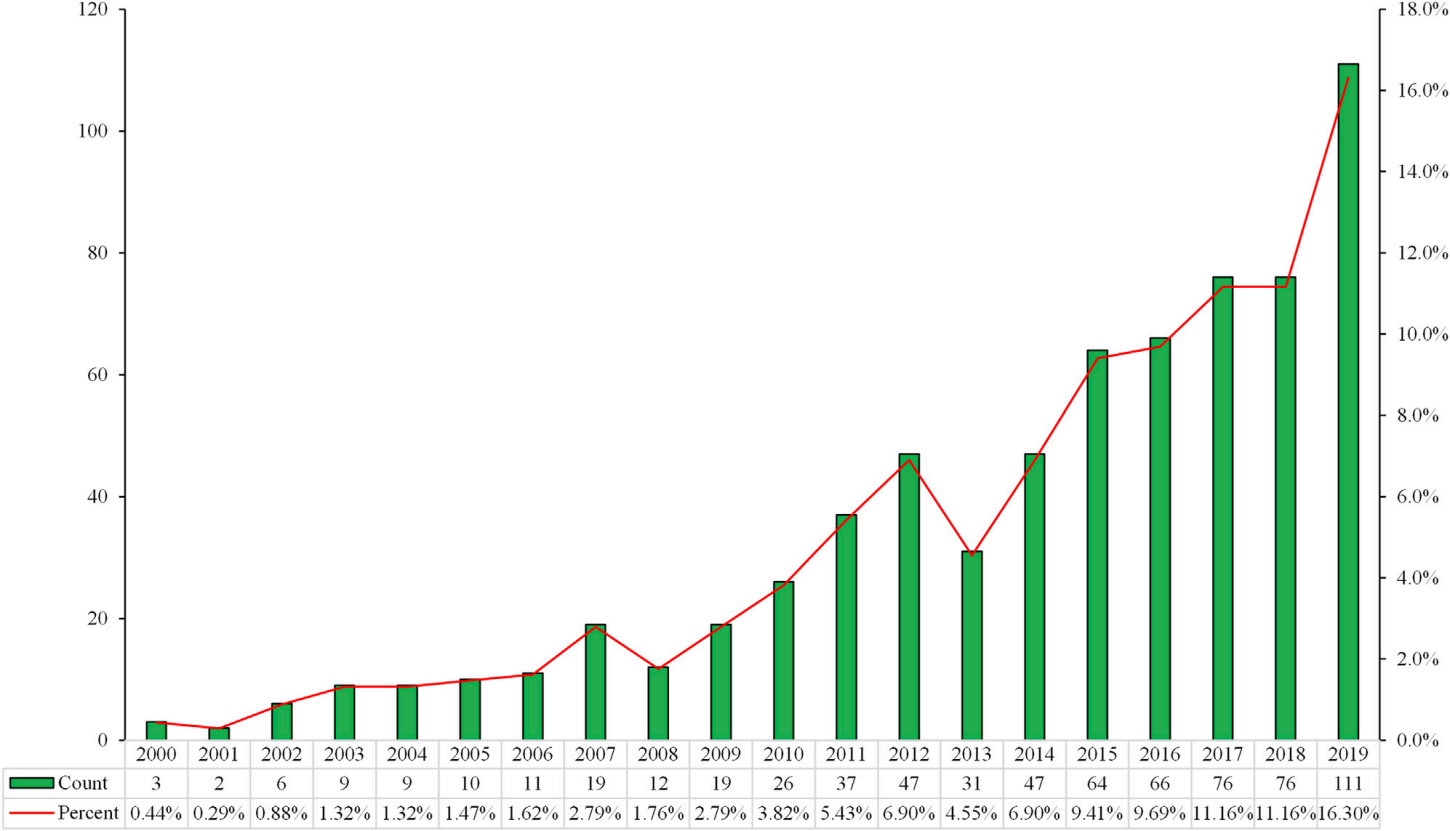
Number of publications per year (2000–2019).

We identified 681 publications associated with PRRT in the WoSCC from 2000 to 2019. Of these 681 publications, 523 (76.80%) were indexed as “article” and 158 (23.20%) as “review.” English was the predominant language for publications on PRRT, constituting 97.06% (661/681) of the total. The most common non-English language was German, which constituted 1.32% (9/681) of the total, followed by French (6, 0.88%), Spanish (3, 0.44%), Hungarian (1, 0.15%), and Polish (1, 0.15%). The annual publication outputs in the PRRT field are shown in Figure 1. The number of publications varied from year to year, with an average of around 34 publications per year represented by an overall upward trend during the investigated period. There were three (0.44%) and two (0.29%) papers published in 2000 and 2001, respectively. The publication number was greater than 20 in 2010, was greater than 60 in 2015, and was the highest in 2019 (*n* = 111, 16.30%).

### Country/Region Analysis

**FIGURE 2:**
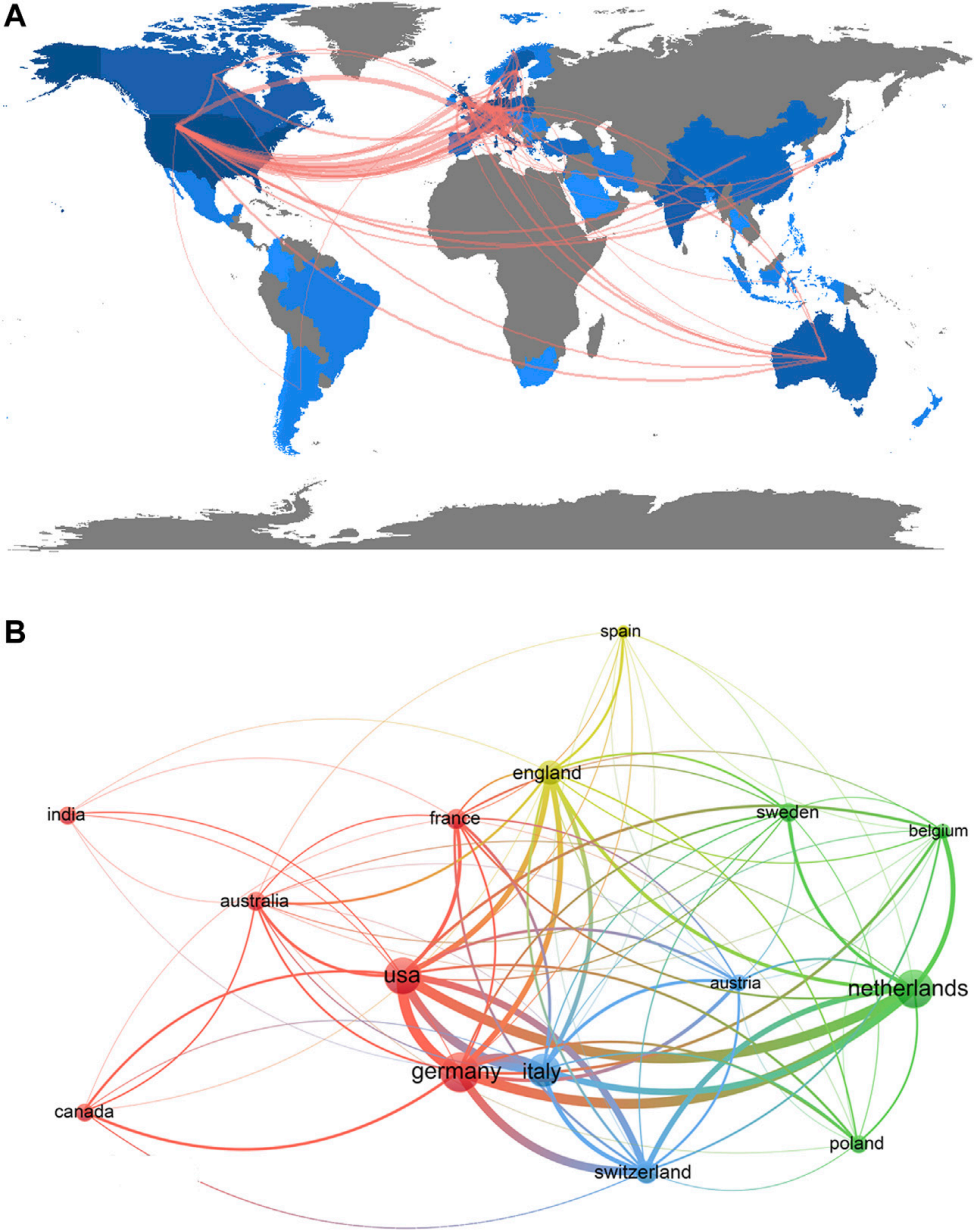
Regional distribution **(A)** and network map of countries/regions (B, T = 15) related to PRRT research.

A total of 55 countries/regions contributed to PRRT research. The top 15 productive countries/regions are shown in Table 1. Germany published the highest number of papers (*n* = 151), followed by the Netherlands (*n* = 142), the United States (*n* = 132), Italy (*n* = 106), England (*n* = 57), Switzerland (*n* = 48), and France (*n* = 39). The top 15 countries/regions were distributed across four continents, of which 11 were located in Europe (Figure 2A). Countries/regions (15/55, 27.27%) with number of publications ≥15 (*T* = 15) were used to construct a country/region co-authorship network (Figure 2B). The network map reflects the state of research activities and communication among these countries/regions. In Figure 2B, Germany, the Netherlands, the United States, and Italy had larger sized bubbles representing higher numbers of papers. There were active collaborations between countries/regions; for example, Germany had close cooperation with the Netherlands, Switzerland, Italy, and the United States.

**TABLE 1:** The top 15 most productive countries/regions for PRRT research.

Rank	Country/region	Count	Rank	Country/region	Count
1	Germany (Europe)	151	9	Australia (Oceania)	33
2	The Netherlands (Europe)	142	10	Poland (Europe)	31
3	The United States (North America)	132	11	India (Asia)	30
4	Italy (Europe)	106	12	Canada (North America)	29
5	England (Europe)	57	13	Austria (Europe)	24
6	Switzerland (Europe)	48	14	Belgium (Europe)	22
7	France (Europe)	39	15	Spain (Europe)	15
8	Sweden (Europe)	34			

### Journal Analysis

**FIGURE 3:**
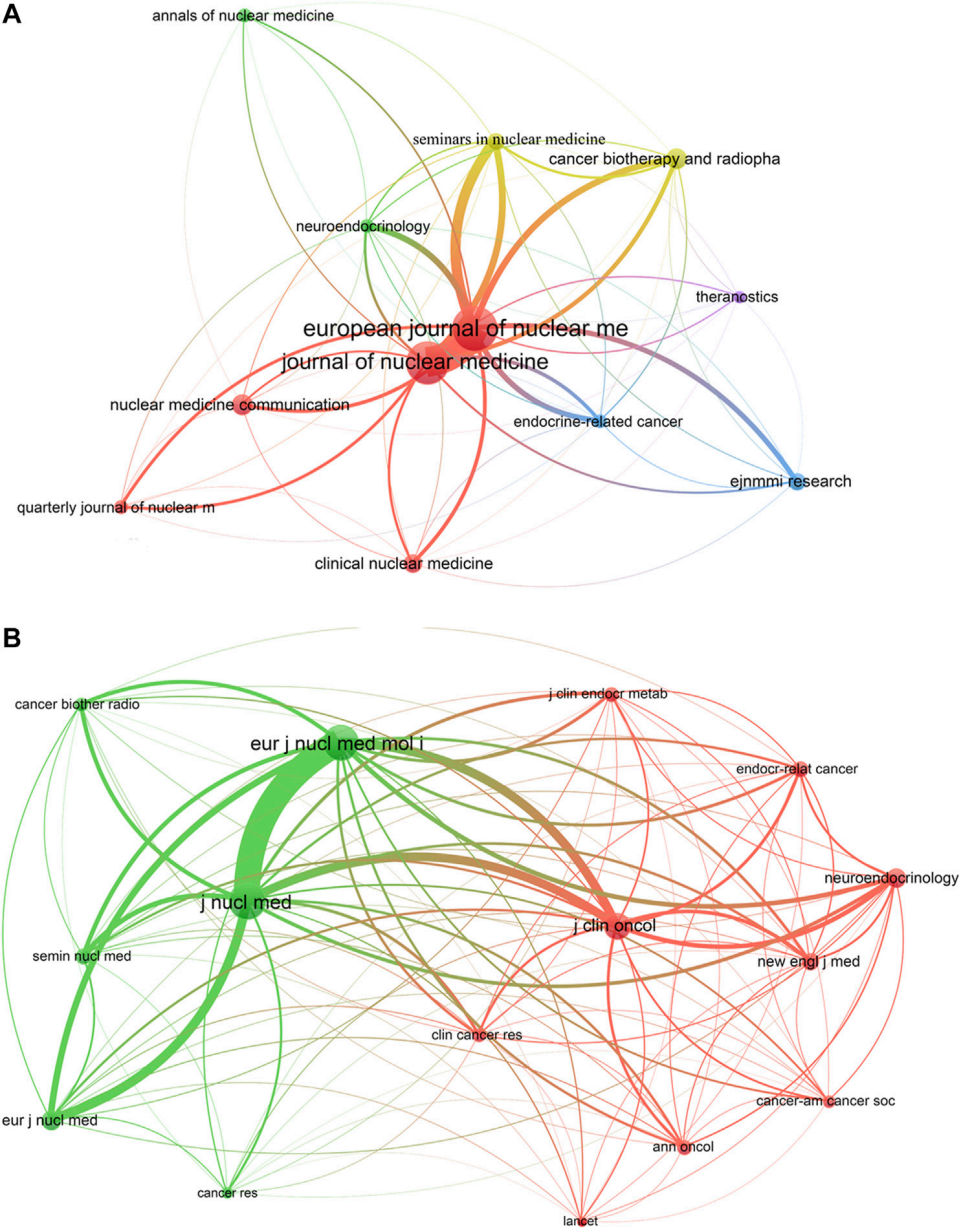
The network map of scholarly journals (A, T = 9) and co-cited scholarly journals (B, T = 241) for PRRT research.

More than 200 scholarly journals (*n* = 214) had published papers on PRRT research. There were four journals with more than 20 publications, of which *European Journal of Nuclear Medicine and Molecular Imaging* (IF2019 = 7.081, Q1) was the most productive journal publishing 84 scientific publications in the field, followed by *Journal of Nuclear Medicine* (*n* = 77, IF2019 = 7.887, Q1), *Cancer Biotherapy and Radiopharmaceuticals* (*n* = 21, IF2019 = 2.314, Q3), and *Nuclear Medicine Communications* (*n* = 21, IF2019 = 1.334, Q4). The top 12 journals with the greatest contribution to PRRT research accounted for 44.35% (302/681) of the total publications included in this study (Table 2). Journals (12/214, 5.61%) with ≥9 publications (*T* = 9) were used to construct the citation network map. As can be seen from Figure 3A, *European Journal of Nuclear Medicine and Molecular Imaging*, *Journal of Nuclear Medicine*, *Cancer Biotherapy and Radiopharmaceuticals*, and *Nuclear Medicine Communications* had larger sized bubbles representing a higher number of publications. *European Journal of Nuclear Medicine and Molecular Imaging* had active citation relationships with *Journal of Nuclear Medicine*, *Seminars in Nuclear Medicine*, and *Cancer Biotherapy and Radiopharmaceuticals*.

**TABLE 2:** The 12 most active journals and co-cited journals that published papers on PRRT research from 2000 to 2019.

Rank	Journal	Count	IF2019	JCR	Co-cited journal	Co-citation	IF2019	JCR
1	*European Journal of Nuclear Medicine and Molecular Imaging* (Germany)	84	7.081	Q1	*Journal of Nuclear Medicine* (The United States)	3,438	7.887	Q1
2	*Journal of Nuclear Medicine* (The United States)	77	7.887	Q1	*European Journal of Nuclear Medicine and Molecular Imaging* (Germany)	3,086	7.081	Q1
3	*Cancer Biotherapy and Radiopharmaceuticals* (The United States)	21	2.314	Q3	*Journal of Clinical Oncology* (The United States)	1769	32.956	Q1
4	*Nuclear Medicine Communications* (The United States)	21	1.334	Q4	European Journal of Nuclear Medicine* (Germany)	975	7.081	Q1
5	*Clinical Nuclear Medicine* (The United States)	17	6.587	Q1	*Neuroendocrinology* (Switzerland)	885	4.271	Q1
6	*Ejnmmi Research* (Germany)	16	2.64	Q2	*New England Journal of Medicine* (The United States)	711	74.699	Q1
7	*Seminars in Nuclear Medicine* (The United States)	15	3.544	Q1	*Endocrine-Related Cancer* (The United States)	589	4.8	Q1
8	*Endocrine-related Cancer* (England)	11	4.8	Q1	*Seminars in Nuclear Medicine* (The United States)	589	3.544	Q1
9	*Neuroendocrinology* (Switzerland)	11	4.271	Q1	*Journal of Clinical Endocrinology and Metabolism* (The United States)	547	5.399	Q1
10	*Annals of Nuclear Medicine* (Japan)	10	2.607	Q2	*Annals of Oncology* (England)	529	18.274	Q1
11	*Quarterly Journal of Nuclear Medicine and Molecular Imaging* (Italy)	10	1.795	Q3	*Clinical Cancer Research* (The United States)	469	10.107	Q1
12	*Theranostics* (Australia)	9	8.579	Q1	*Cancer Biotherapy and Radiopharmaceuticals* (The United States)	445	2.314	Q3

IF, impact factor; JCR, journal citation reports; Q, quartile in category.

* *European Journal of Nuclear Medicine* is now called *European Journal of Nuclear Medicine and Molecular Imaging*.

When two journals are cited simultaneously in one or more publications, the two journals have a co-citation relationship (Ke et al., 2020). There were 2,269 co-cited scholarly journals, and three journals had over 1,000 co-citations, of which two were issued by the United States and one by Germany (Table 2). *Journal of Nuclear Medicine* had the most co-citations (*n* = 3,438, IF2019 = 7.887, Q1), followed by *European Journal of Nuclear Medicine and Molecular Imaging* (*n* = 3,086, IF2019 = 7.081, Q1) and *Journal of Clinical Oncology* (*n* = 1769, IF2019 = 32.956, Q1) (Table 2). Journals (15/2,269, 0.66%) with co-citations ≥ 241 (*T* = 241) were used to construct the co-citation network. As shown in Figure 3B, *Journal of Nuclear Medicine* and *European Journal of Nuclear Medicine and Molecular Imaging* had larger bubbles due to higher co-citations, and *Journal of Nuclear Medicine* had active co-citation relationships with *European Journal of Nuclear Medicine and Molecular Imaging* and *Journal of Clinical Oncology*.

**FIGURE 4:**
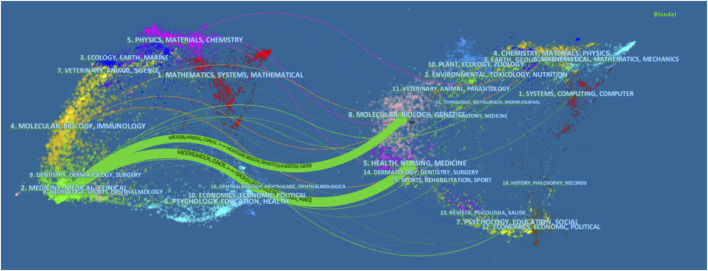
The dual-map overlay of journals related to PRRT research.

A dual-map overlay of journals was displayed using CiteSpace (Figure 4). The citing and cited journal maps are the left and the right cluster, respectively. The lines that start from the left to the right are citation links (Ke et al., 2020). We found that there were two main citation paths (green). The top green path indicates that papers published in Medicine/Medical/Clinical journals usually cited papers published in Molecular/Biology/Genetics journals, while the bottom green path shows that papers published in Medicine/Medical/Clinical journals primarily cited papers published in Health/Nursing/Medicine journals.

### Reference Analysis

**FIGURE 5:**
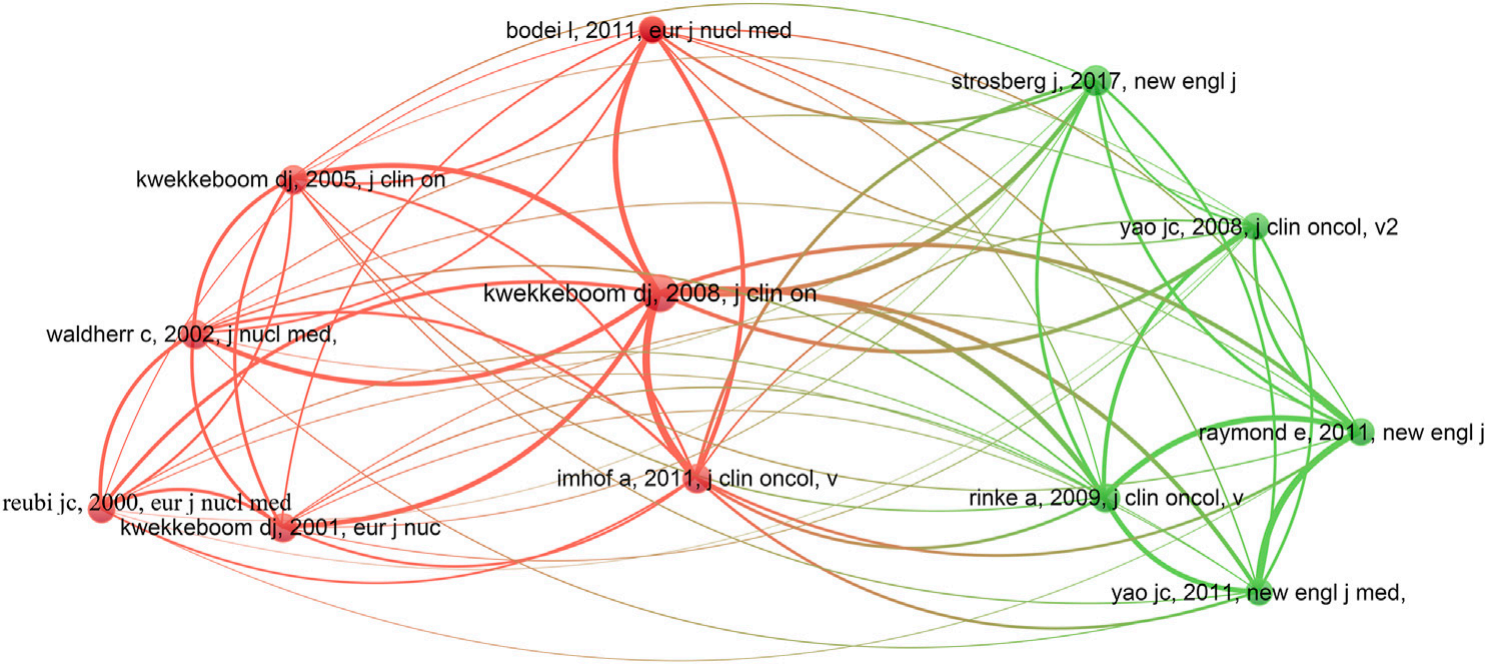
Co-citation map of references (T = 102) from publications on PRRT research.

Co-cited references are references that have been co-cited in a set of publications (Ke et al., 2020; Hou et al., 2018). The top 12 co-cited references are listed in Table 3. Each reference of the top 12 co-cited references was co-cited at least 102 times. Among them, a publication by Kwekkeboom et al. published in *Journal of Clinical Oncology* had the highest number of co-citations (2008, 254 citations) (Kwekkeboom et al., 2008), followed by article published by Strosberg et al. (2017, 140 citations) in *New England Journal of Medicine* (Strosberg et al., 2017), Imhof et al. (2011, 134 citations) in *Journal of Clinical Oncology* (Imhof et al., 2011), and Rinke et al. (2009, 125 citations) in *Journal of Clinical Oncology* (Rinke et al., 2009). The number of co-citations in the remaining eight references ranged from 102 to 121. References (12/12,747, 0.09%) with co-citations ≥ 102 (*T* = 102) were used to construct the co-citation map. As shown in Figure 5, “Kwekkeboom DJ, 2008, *Journal of Clinical Oncology* (Kwekkeboom et al., 2008)” had the largest size and had active co-cited relationships with “Imhof A, 2011, *Journal of Clinical Oncology* (Imhof et al., 2011)” and “Bodei L, 2011, *European Journal of Nuclear Medicine and Molecular Imaging* (Bodei et al., 2011).”

**TABLE 3:** The top 12 co-cited references in PRRT research.

Rank	Co-cited reference	Count
1	Kwekkeboom et al. (2008), *Journal of Clinical Oncology*, V26, P2124	254
2	Strosberg et al. (2017), *New England Journal of Medicine*, V376, P125	140
3	Imhof et al. (2011), *Journal of Clinical Oncology*, V29, P2416	134
4	Rinke et al. (2009), *Journal of Clinical Oncology*, V27, P4656	125
5	Waldherr et al. (2002), *Journal of Nuclear Medicine*, V43, P610	121
6	Kwekkeboom et al. (2001), *European Journal of Nuclear Medicine*, V28, P1319	118
7	Kwekkeboom et al. (2005), *Journal of Clinical Oncology*, V23, P2754	116
8	Reubi et al. (2000), *European Journal of Nuclear Medicine*, V27, P273	112
9	Yao et al. (2008), *Journal of Clinical Oncology*, V26, P3063	109
10	Raymond et al. (2011), *New England Journal of Medicine*, V364, P501	108
11	Yao et al. (2011), *New England Journal of Medicine*, V364, P514	105
12	Bodei et al. (2011), *European Journal of Nuclear Medicine and Molecular Imaging*, V38, P2125	102

### Burstness Analysis

**FIGURE 6:**
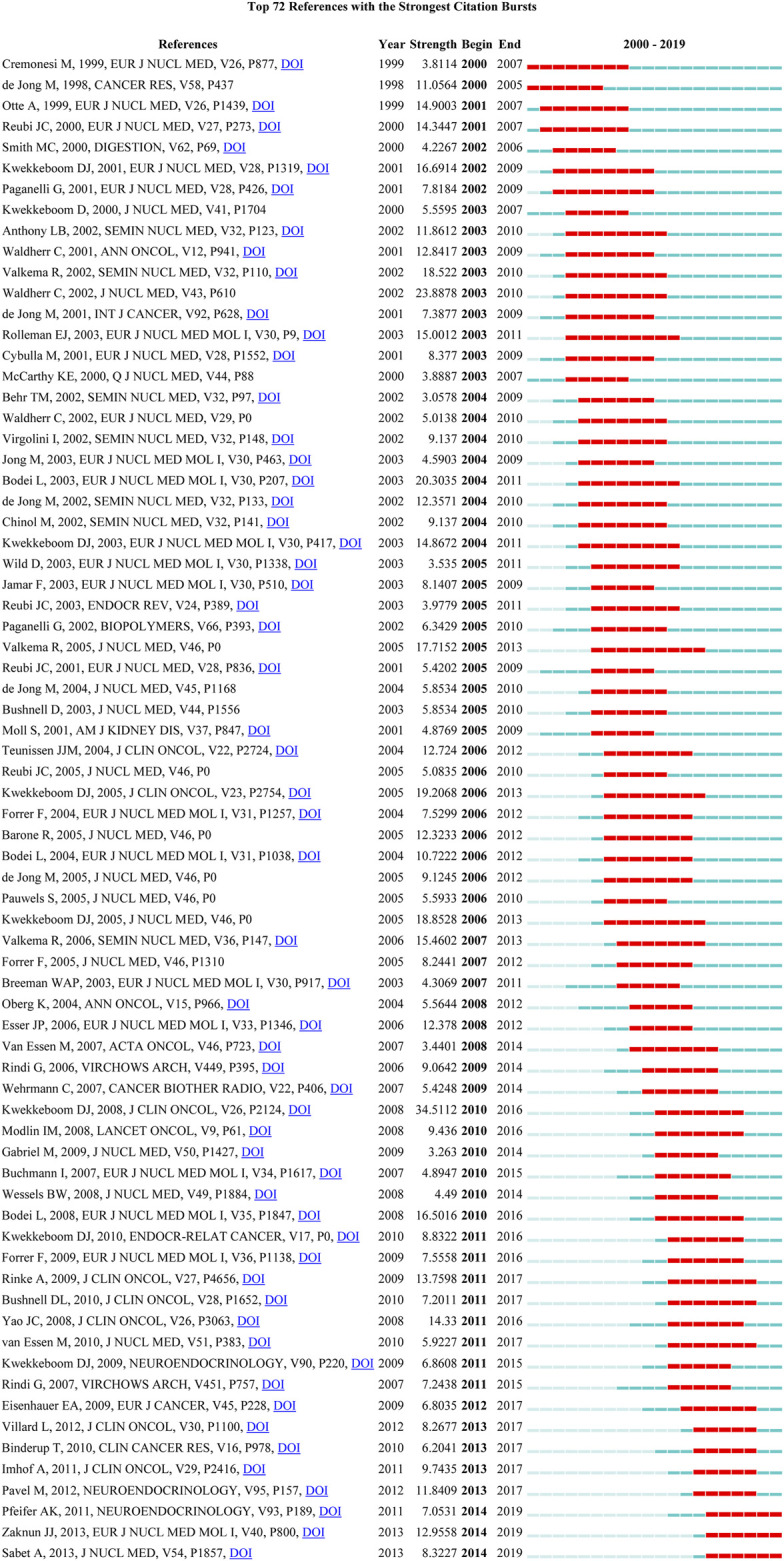
The references with strong citation burstness (MD = 5) of publications related to PRRT research published from 2000 to 2019.

Burstness detection allows the identification of publications that receive particular attention from related scientific communities during a certain period of time (Ke et al., 2020; Hou et al., 2018). In CiteSpace, the minimum duration of burstness was set to five years in the present research, and 72 references with strong citation burstness were detected (Figure 6). In Figure 6, the length of the line represents the period from 2000 to 2019, in which the red line indicates the time interval of citation burstness. Among the 72 references, citation burstness of 20 references ended in 2015 or later. The strongest burstness (strength = 34.5112) among the 20 references was the article entitled “Treatment with the radiolabelled somatostatin analog [^177^Lu-DOTA^0^,Tyr^3^] octreotate: toxicity, efficacy, and survival” published in *Journal of Clinical Oncology* by Kwekkeboom et al. with citation burstness lasting from 2010 to 2016 (Kwekkeboom et al., 2008), followed by “Long-term evaluation of renal toxicity after peptide receptor radionuclide therapy with 90Y-DOTATOC and 177Lu-DOTATATE: the role of associated risk factors” published by Bodei et al. with citation burstness lasting for seven years (2010–2016, strength = 16.5016) (Bodei et al., 2008). Special attention should be paid to three references that had citation burstness ending in 2019: “The joint IAEA, EANM, and SNMMI practical guidance on peptide receptor radionuclide therapy (PRRNT) in neuroendocrine tumors” published by Zaknun et al. had the strongest citation burstness (strength = 12.9558) (Zaknun et al., 2013), followed by papers published by Sabet et al. (strength = 8.3227) in *Journal of Nuclear Medicine* (Sabet et al., 2013) and Pfeifer et al. (strength = 7.0531) in *Neuroendocrinology* (Pfeifer et al., 2011). The citation burstness of these three papers lasted from 2014 to 2019.

## Discussion

### Basic Information

Based on the literature from 2000 to 2019 relating to PRRT research from the SCIE of the WoSCC, we performed a bibliometric analysis to gain a comprehensive view of the research trends concerning PRRT during the past two decades and to provide references for researchers in this field. The number of scientific publications reflects the speed of development of a specific research area (Ke et al., 2020). Our research found that three papers were published in 2000 and only two in 2001, which may be related to the fact that the field was still in its infancy. Indeed, the clinical study with ^90^Y-dotatoc was started in Basel in June 1996, and the first clinical studies with ^177^Lu-dotadate started in 2000 in Rotterdam, the Netherlands (Levine and Krenning, 2017). The number of annual publications showed significant increases in recent years, especially from 2015, when more than 60 papers were published. Overall, the annual output related to PRRT research followed an upward trend during the investigated period, which suggested that PRRT research has received increasing attention in recent years. Germany, the Netherlands, and the United States were the top three productive countries, two of which are located in Europe and one in North America, demonstrating that these three countries are particularly influential in the field of PRRT research. Among the top 15 countries, there was only one Asian country (India), which indicated that the research capacity of Asian countries/regions in this field is relatively weak. As for journals, *European Journal of Nuclear Medicine and Molecular Imaging*, the official journal of the European Association of Nuclear Medicine, was the influential journal in the field, which ranked the first in the productive journals and second in the co-cited journals. In addition to *European Journal of Nuclear Medicine and Molecular Imaging*, there were five other journals that were both top productive journals and top co-cited journals: *Journal of Nuclear Medicine*, *Endocrine-related Cancer*, *Neuroendocrinology*, *Seminars in Nuclear Medicine*, and *Cancer Biotherapy and Radiopharmaceuticals*. Journals issued by the United States accounted for the largest proportion of the top 12 active journals (41.67%) and top 12 co-cited journals (58.33%), which indicated that the United States has considerable influence in this field. The dual-map overlay of journals analysis may provide a reference for beginners to conduct research in the field of PRRT. As for scholars in the PRRT research field, in the top 12 co-cited references, Kwekkeboom from Erasmus Medical Center published three papers (Kwekkeboom et al., 2001; Kwekkeboom et al., 2005; Kwekkeboom et al., 2008), indicating that this author made a significant contribution and may be regarded as the representative author in the area of PRRT. Next, we used the co-cited references to find the knowledge base related to PRRT and discussed in the following section.

### Knowledge Base

Co-cited references represent how frequently two publications are cited together by other publications and may be regarded as a knowledge base of a specific field or subject (Ke et al., 2020). In our study, the top 12 co-cited references were selected to identify the knowledge base related to PRRT. The study with the highest number of co-citations (*n* = 254) was published in 2008 by Kwekkeboom et al. (Kwekkeboom et al., 2008), which demonstrated complete or partial tumor remissions in patients with metastasized or inoperable gastroenteropancreatic NETs (GEP-NETs) treated with ^177^Lu-dotatate. The preliminary results of this treatment in relatively small patient groups were also reported previously by Kwekkeboom et al. Two of these belonged to the top 12 co-cited references, specifically the sixth and seventh co-cited references (*n* = 118 and *n* = 116) published in 2001 (Kwekkeboom et al., 2001) and 2005 (Kwekkeboom et al., 2005), respectively. In 2001, Kwekkeboom et al. found that the ^177^Lu-dotatate represents a potentially important improvement because the uptake of radioactivity was comparable to that after [^111^In-DTPA^0^] octreotide in the organs (e.g., kidneys, spleen, and liver) but was three- to fourfold higher in most tumors. In 2005, Kwekkeboom et al. presented the preliminary results of treatment with ^177^Lu-dotatate in 131 patients with GEP-NETs showing tumor remission in a high percentage of patients. The second co-cited publication (*n* = 140) was published in the *New England Journal of Medicine* by Strosberg et al. (Strosberg et al., 2017). Approval of Lutathera® (^177^Lu-dotatate) in the United States and Europe was primarily based on the positive results of this multinational phase III clinical trial, which compared ^177^Lu-dotatate treatment with high doses of long-acting release octreotide. In 2011, Imhof et al. published the third co-cited reference in *Journal of Clinical Oncology* (*n* = 134). In this clinical phase II trial, patients with neuroendocrine cancers were treated with repeated cycles of ^90^Y-dotatoc. The results showed morphologic response and longer survival in 34.1 and 79% of the patients, respectively; meanwhile, hematologic and renal toxicity was reported in a small number of patients (Imhof et al., 2011). *Journal of Clinical Oncology* published the fourth most co-cited study by Rinke et al. (Rinke et al., 2009). Unlike the above co-cited references, which evaluated the efficacy and/or safety of PRRT, this study provided evidence that long-acting release octreotide inhibits tumor growth in patients with metastatic well-differentiated midgut NETs. Waldherr et al. published the fifth most co-cited study in 2002, with 121 co-citations. This study evaluated the tumor response to high-dose targeted irradiation with 7.4 GBq/m^2^ of ^90^Y-dotatoc in patients with NETs. Compared with the 6 GBq/m^2^ dose used in a previous study, the 7.4 GBq/m^2^ dose of ^90^Y-dotatoc was well tolerated as a treatment for NETs (Waldherr et al., 2002). The eighth co-cited reference was published by Reubi et al. in 2000. Using cell lines transfected with somatostatin receptor subtypes 1 to 5, Reubi and colleagues evaluated the *in vitro* binding characteristics of radiolabeled (indium, yttrium, and gallium) and unlabeled somatostatin analogs. One of the results demonstrated *in vitro* that the somatostatin analog [DOTA^0^,Tyr^3^] octreotate had an approximately nine fold higher affinity for somatostatin receptor subtype 2 than [DOTA^0^,Tyr^3^] octreotide. The authors believed that these observations might represent basic principles relevant to the development of other peptide radioligands (Reubi et al., 2000). Yao et al. published the ninth co-cited reference in 2008 (Yao et al., 2008), which examined the epidemiology of NETs and prognostic factors for NETs. The authors observed an increasing trend for incidence of NETs, and histologic grade, primary tumor site, disease stage, etc., were prognostic factors of outcome. Additionally, the authors mentioned some new therapeutic approaches for NETs at the end of the paper, such as PRRT and targeted agents. Similar to the long-acting release octreotide reported in the fourth most co-cited reference (Rinke et al., 2009), two other therapies considered for the treatment of NETs were evaluated in the tenth and eleventh most co-cited articles (Raymond et al., 2011; Yao et al., 2011). They were frequently cited in literature on PRRT treatment of NETs. *New England Journal of Medicine* published the tenth most commonly co-cited study by Raymond et al. in 2011, an international phase III study of sunitinib vs. placebo in patients with progressive well-differentiated endocrine pancreatic tumor. The results showed that sunitinib significantly reduced the risk of disease progression and led to a prolongation of progression-free survival by 5.6 months (11.1 vs. 5.5 months) compared to placebo (Raymond et al., 2011). The eleventh most commonly co-cited article was published by Yao et al. in *New England Journal of Medicine* in 2011. This large international placebo-controlled trial evaluated the efficacy of everolimus in 410 patients with progressive pancreatic NET. The risk of disease progression was significantly reduced due to the striking superiority of everolimus, which was demonstrated by a progression-free survival of 11 vs. 4.6 months compared to placebo (Yao et al., 2011). The article published in *European Journal of Nuclear Medicine and Molecular Imaging* in 2011 received the last co-citations of the top 12, which observed that ^177^Lu-dotatate was well tolerated up to 29 GBq cumulative activity (up to 7.4 GBq/cycle). However, considering the bone marrow function of patients and the presence of risk factors for kidney toxicity, it seemed safer to divide cumulative activities into lower activity cycles (Bodei et al., 2011). Based on the top 12 co-cited references, we found that the knowledge base of PRRT research mainly involved the following aspects: efficacy of PRRT (e.g., ^90^Y-dotatoc and ^177^Lu-dotatate), adverse effects (e.g., hematologic and renal toxicities) of PRRT, epidemiologic characteristics, and other therapies (e.g., somatostatin analogs and targeted agents) for NETs (e.g., GEP-NETs).

### Emerging Topics

Papers with high citation burstness can, to a certain extent, reflect the emerging trends or topics within a field (Hou et al., 2018; Ke et al., 2020). The top 72 references with strong strength citation burstness were identified using CiteSpace; the citation burstness of 20 of them ended in 2015 or later, reflecting the most recent topics in PRRT research; these were selected for further discussion. In regard to the end times for citation burstness, three of these 20 references ended in 2015, six in 2016, eight in 2017, and three in 2019. Based on the research content, six of these 20 references evaluated the efficacy and/or safety of PRRT with a single radioisotope (^177^Lu or ^90^Y) or a combination of radioisotopes (^177^Lu and ^90^Y) (Kwekkeboom et al., 2008; Bushnell et al., 2010; van Essen et al., 2010; Imhof et al., 2011; Pfeifer et al., 2011; Villard et al., 2012). Among them, the strongest burstness was due to an article published by Kwekkeboom et al. in 2008 (strength = 34.5112), which had a citation burstness that lasted for six years (2011–2016) (Kwekkeboom et al., 2008). The publication with the second highest citation burstness was published in *Journal of Clinical Oncology* by Imhof et al. in 2011. It had a burstness strength of 9.7435, and the burstness lasted for 5 years (2013–2017) (Imhof et al., 2011). Simply put, these two studies showed encouraging outcomes in patients with NETs treated with ^177^Lu-dotatate or ^90^Y-dotatoc. The article with the third highest citation burstness (strength = 8.2677) published by Villard et al. in 2012 evaluated the efficacy of PRRT with ^90^Y-dotatoc vs. ^90^Y-dotatoc plus ^177^Lu-dotatoc in patients with NETs, and results showed that the combination of radioisotopes was associated with improved overall survival compared with a single radioisotope (Villard et al., 2012). The fourth highest citation burstness (strength = 7.2011) was a study conducted by Bushnell et al. in 2010, and its burstness lasted for seven years (2011–2017). The results revealed that ^90^Y-dotatoc treatment improved symptoms associated with malignant carcinoids in patients with no treatment alternatives (Bushnell et al., 2010). *Neuroendocrinology* published the fifth highest citation burstness study of the six references by Pfeifer et al. in 2011. This retrospective study evaluated the treatment responses and adverse effects of PRRT with ^177^Lu-dotatate and ^90^Y-dotatoc in patients with NETs, and the authors concluded that the implementation of PRRT provides a valuable new therapeutic option in the treatment of advanced NETs (Pfeifer et al., 2011). Finally, the publication with the sixth highest citation burstness was published by van Essen et al. in 2010, with a burstness strength of 5.9227 that lasted from 2011 until 2017. This study showed that salvage therapy with ^177^Lu in patients with bronchial and GEP-NETs is effective and safe (van Essen et al., 2010). Five of these 20 references provided guidelines and criteria; among them, the paper with the strongest burstness was a guideline published by Zaknun et al. in 2013 (strength = 12.9558), for which the citation burstness lasted for six years (2014–2019) (Zaknun et al., 2013). The guidance was formulated through a joint international effort under the auspices of the International Atomic Energy Agency, in cooperation with the European Association of Nuclear Medicine and the Society of Nuclear Medicine and Molecular Imaging. It covered the rationale, indications, and contraindications for PRRT; assessment of treatment response; and patient follow-up, and was aimed at guiding nuclear medicine specialists in selecting likely candidates for receiving PRRT and delivering the treatment in a safe and effective manner. The theme of one guideline (Kwekkeboom et al., 2009) issued by European Neuroendocrine Tumor Society in 2009 was similar to the above-mentioned guideline. Moreover, another guideline issued by this society in 2012 was for the management of patients with liver and other distant metastases from neuroendocrine neoplasms of the foregut, midgut, hindgut, and unknown primary (Pavel et al., 2012), where PRRT was discussed as one of the treatment approaches. The remaining two papers provided evaluation criteria and focused on tumor-node-metastasis staging of midgut and hindgut (neuro) endocrine tumors and the response evaluation criteria in solid tumors (Rindi et al., 2007; Eisenhauer et al., 2009). The former aimed to help clinicians find the most suitable treatment for patients at different stages of tumors, such as recommending surgery when complete resection is possible and medical treatment (e.g., somatostatin analogs, interferon, chemotherapy, PRRT, and targeted agents) when tumors are unresectable. The latter offered recommendations on the assessment of treatment outcomes. Of the 20 references, two studies focused on the long-term adverse effects of PRRT. One study published by Sabet et al. in 2013 with burstness that lasted six years (2014–2019) investigated the incidence, severity, and reversibility of long-term hematotoxicity in a large cohort of patients undergoing PRRT with ^177^Lu-dotatate for metastatic NETs (Sabet et al., 2013). The second study published by Bodei et al. in 2008 investigated the long-term behavior of the main parameters of renal function in a subgroup of patients treated with ^90^Y-dotatoc or ^177^Lu-dotatate in the past decade (Bodei et al., 2008). Given that PRRT is a relatively new method, only a small number of long-term follow-up studies focusing on toxicity after PRRT have been published to date, and thus more prospective studies investigating these aspects are needed. Two publications were reviews about GEP-NETs (Modlin et al., 2008; Kwekkeboom et al., 2010). The study by Modlin et al. in 2008 reviewed GEP-NETs from biological and clinical perspectives and systematically introduced the treatment of GET-NETs. They provided recommendations for current scientific and clinical limitations (Modlin et al., 2008). The other review focused on somatostatin receptor-based imaging and therapy of GEP-NETs (Kwekkeboom et al., 2010). Two articles focused on the use of medical imaging techniques in NETs. As patients with NETs are a very heterogeneous group in terms of symptoms, clinical course, and treatment strategy, imaging modalities could be helpful in selecting the appropriate treatment for each patient (Buchmann et al., 2007; Binderup et al., 2010). One study published by Forrer in 2009, of which the citation burstness lasted for six years (2011–2016), reported that the individual calculation of the bone marrow absorbed dose is necessary for individual dose optimization (Forrer et al., 2009). Individualized and accurate treatment is the trend for PRRT because the current dosing paradigm for Lutathera^®^ may overtreat some patients, where patients may not require the full four cycles of therapy. The study published by Yao et al. (2008) examined the epidemiology of and prognostic factors for NETs, and the study published by Rinke et al. (2009) evaluated the efficacy of long-acting release octreotide in patients with metastatic well-differentiated midgut NETs. In summary, through in-depth discussion of the 20 references with citation burstness ending in 2015 or later, we found that the most prominent topics for PRRT included matters related to the efficacy (e.g., ^90^Y-dotatoc, ^177^Lu-dotatate, and ^177^Lu-dotatoc), long-term adverse effects (e.g., hematologic and renal toxicities), standardization of NETs and PRRT in practice, development of medical imaging techniques, and individual dose optimization.

### Strengths and Limitations

Our study has several strengths. First, the present study provided a deep insight into the global status and trends of research on PRRT using bibliometric analysis for the first time. Second, widely used tools were applied in our study, which assures the reliability of the data. Third, compared with traditional literature reviews, bibliometric analysis is relatively more objective and comprehensive. Nevertheless, this study should also be considered in light of several limitations, which are similar to other bibliometric analyses. First, we only surveyed publications in the WoSCC database; other databases, such as PubMed, Embase, and Cochrane library, were not searched, which may result in some omitted publications. However, it should be noted that the WoSCC is the most frequently used database for bibliometric analysis (Yang et al., 2018; Ke et al., 2020). Second, differences may exist between the bibliometric analysis results and real-world research conditions. The results of our study came from published research but some important information may not have been published in the form of scientific publications. Third, some recently published important papers might not have gained enough attention from researchers and thus may not be discussed in detail in our study. For example, the review (Nicolas et al., 2019) recommended by a reviewer, which is a review published in 2019, summarized the recent developments (e.g., ^213^Bi and ^225^Ac) in PRRT. Therefore, there is still a need to observe the latest published achievements. Finally, all information was extracted using tools, so there are some issues that we encountered in our study that need attention (e.g., journals changing their names affected our results to some extent). Despite these limitations, this study provides a solid global view on PRRT research from the last two decades.

## Conclusion

We used bibliometric analysis to provide a comprehensive overview of the research status of PRRT worldwide during the past two decades. The PRRT field is undergoing a period of rapid development and attracting increased attention from researchers. Germany ranked first for productivity and closely cooperated with other countries, such as the Netherlands and the United States. Kwekkeboom from Erasmus Medical Center is perhaps a key researcher in the field of PRRT. *European Journal of Nuclear Medicine and Molecular Imaging* and *Journal of Nuclear Medicine* ranked first in the productive journals and co-cited journals, respectively. The key research topics identified in our study included the efficacy and safety of PRRT, standardization of NETs and PRRT in practice, the development of medical imaging techniques, and individual dose optimization of PRRT. The topics regarding PRRT deserve continued following up by researchers, and we believe that our study provides a valuable reference for clinical researchers and practitioners.

## Data Availability

The original contributions presented in the study are included in the article/Supplementary Material. Further inquiries can be directed to the corresponding authors.
